# Supplementary material for: dTOURS: Dense-region tagging for outbreak detection using ratio statistics
Source: PLoS One. 2025 May 13;20(5):e0322663. doi: 10.1371/journal.pone.0322663 (PMC12074585; doi:10.1371/journal.pone.0322663)
Supplement: Attachment — Submitted filename: rebuttal_letter.docx [file pone.0322663.s008.docx]

Dear Academic editor and Reviewers:

Thank you for your reports on our article, "*dTOURS: dense-region tagging for outbreak detection using ratio statistics*”. We have addressed all comments as described below. We look forward to receiving any additional comments you may have on this revision of the article.

Thank you,

Richa Agarwala

Remarks to the Academic editor:

We have removed the phrase “data not shown” and added the following example on lines 202-206 of the manuscript without track changes:

An example is SNP calls on SKESA assemblies of SRR3657301 and SRR3747657 where the SKA alignment for a region with a SNP has a 32 bp alignment flanked by 47 and 60 gaps on 5’ and 3’ ends. The 32 bp sequence in the alignment from SRR3657301 (CGTCATACTCGACGCCGTTAATCACCGCCTGT) has matches for the first and last 17 bases to two different contigs with no longer alignment. This illustrates that the 32 bp sequence is not supported by any contig in SRR3657301.

Remarks to Reviewer 1:

1. Include a summary figure of the dTOURS algorithm to give an overview of the approach and enhance the paper's readability.

We have added Fig 1 that presents an overview of the approach and clarified the text. We moved the implementation of sorted lists from the section on “dTOURS implementation for fragmented genomes” to the “dTOURS algorithm” as the implementation holds true for all inputs and clarifies how the compute is done.

2. Provide results for Gubbins on simulated data for comparison. If not already conducted, please include these results.

Results for both Gubbins and FDA method on simulated data have been added. However, the time taken by Gubbins is orders of magnitudes more than dTOURS for millions of pairs, so we subsampled input down to 25,000 sets out of one million for each of the 35 combinations for the comparison.

3. Clarify the color coding used in Tables 1-3.

We moved Table 1 and 3 to Supplementary information as the font was becoming too small and inconsistent between tables. The color coding for both these tables has been added to caption. Both tables color code cells in the table using 2-color scale from green to red where small values have cells colored green and cells with large values have red color. For Table 2, we added “Each value in the table has a different color to make it easy to see patterns in the data.”

4. Ensure consistent font sizes across Table 1 and Table 2 (e.g., "Clade 3" appears unequal).

Table 1 has been moved to Supplementary information.

5. While the paper focuses on pathogen outbreaks, like Gubbins, dTOURS likely has broader applications. It will benefit the community if the discussion includes potential additional applications of dTOURS.

We added following in the discussion:

“dTOURS has potential to be used in other contexts for identifying the existence and boundaries of clusters of discrete events, though the only application we have explored to date is point differences among bacterial isolates. For example, the distribution of RNA editing sites in Alu and LINE elements is known to have roughly lognormally variable local density [32]. Rough estimation using ad-hoc density thresholds [32,33] is a common approach to identifying and characterizing these clusters. Computational implementation of a probabilistic basis in dTOURS might be useful for analyses of these events in Alu or LINE elements.”

Remarks to Reviewer 2:

1. refining the explanation of the ratio statistic

Same as comment 1 of Reviewer 1

2. incorporating a computational efficiency analysis

This was added as part of comparison suggested in comment 2 of Reviewer 1.

3. expanding comparisons with additional SNP detection tools beyond Gubbins

This was added as part of comparison suggested in comment 2 of Reviewer 1. Second method we used is the method used by the Food and Drug Administration.

4. Enhancing result visualization with annotated figures and addressing potential false positives and negatives

We enhanced analysis for the first pair in section on “Comparison to Gubbins” and added Fig 5 to visually show regions removed by both dTOURS and Gubbins and SNPs only removed by dTOURS on the same contig. The figure also shows alignment of the entire contig to a known plasmid.
